# Safety and effectiveness of guselkumab in Japanese patients with psoriasis: 20‐week interim analysis of a postmarketing surveillance study

**DOI:** 10.1111/1346-8138.17255

**Published:** 2024-05-15

**Authors:** Yayoi Tada, Yukako Sugiura, Manami Kamishima, Yoshihito Tanaka, Hiroaki Tsuchiya, Junya Masuda, Keiichi Yamanaka

**Affiliations:** ^1^ Department of Dermatology Teikyo University School of Medicine Tokyo Japan; ^2^ Janssen Pharmaceutical K.K. Tokyo Japan; ^3^ Department of Dermatology Mie University Graduate School of Medicine Tsu Japan

**Keywords:** biologics, guselkumab, interleukin 23 subunit p19, psoriasis, real world

## Abstract

A 52‐week postmarketing surveillance study was initiated to evaluate the safety and effectiveness of guselkumab, a human anti–interleukin 23 subunit p19 monoclonal antibody, in Japanese patients with psoriasis vulgaris, psoriatic arthritis, generalized pustular psoriasis, and erythrodermic psoriasis in real‐world practice. Here, we report results of the 20‐week interim analysis of the ongoing postmarketing surveillance study. Patients who received guselkumab between May 2018 (the date of commercial launch in Japan) and October 2020 were registered in this study. In total, 411 and 245 patients were included in the safety and effectiveness analysis sets, respectively. Adverse drug reactions (ADRs) occurred in 6.6% (27 of 411) and serious ADRs in 2.2% (nine of 411) of patients. The most frequent ADRs by System Organ Class were “Infections and infestations” (2.4%), with nasopharyngitis being the most frequently observed ADR (0.7%). The mean Psoriasis Area Severity Index score decreased from 11.6 at baseline to 6.5 at week 4 and 2.2 at week 20, with improvements achieving statistical significance at each time point. Clinical Global Impression, Dermatology Life Quality Index, and Nail Psoriasis Severity Index outcomesalso showed substantial improvements. Our findings demonstrate that guselkumab is well tolerated and effective in Japanese patients with psoriasis through 20 weeks of treatment in real‐world clinical practice, showing significant effectiveness observed as early as 4 weeks. The study was officially registered with the University Hospital Medical Information Network Clinical Trials Registry with the identifier UMIN000032969.

## 1 INTRODUCTION

Psoriasis is a chronic, immune‐mediated skin disorder that may lead to adverse health outcomes.^1^ Particularly in advanced stages, psoriasis is associated with various comorbidities, which also have a negative impact on patients' quality of life.^1^, ^2^, ^3^, ^4^

Conventional treatments for psoriasis include topical drugs, oral immunosuppressants, and phototherapies. In addition, there are more than 10 biologics that target various cytokines, along with new small‐molecule therapeutics such as phosphodiesterase‐4, Janus kinase, and tyrosine kinase 2 inhibitors, for treating psoriasis with cytokine‐driven inflammation. In Japan, the first biologics for psoriasis were infliximab and adalimumab (anti–tumor necrosis factor α [TNF‐α] antibodies) introduced in 2010, followed by ustekinumab (anti–interleukin 12/IL‐23 subunit 40 [IL‐p40] antibody) in 2011. Since 2015, a variety of other biologics have become available including secukinumab and ixekizumab (anti–IL‐17A antibodies), brodalumab (anti–IL‐17R antibody), guselkumab (GUS), risankizumab and tildrakizumab (anti–IL23p19 antibodies), certolizumab pegol (anti–TNF‐α antibody Fab fragment), and bimekizumab (anti–IL‐17A/F antibody). These treatments provide patients and physicians with an expanded range of therapeutic options for psoriasis.^5^, ^6^, ^7^, ^8^ The Japanese guidance on the use of biologics for the treatment of psoriasis from the Japanese Dermatological Association (JDA) states that it is important for physicians to select appropriate biologic therapy for each patient with psoriasis after due consideration of disease factors, treatment factors, and patient background factors, sharing such information with patients. The JDA guidance also notes that when selecting biologics, factors to be considered include not only the drug's effectiveness (such as likelihood of high level response, time to onset of effectiveness, effectiveness against arthritis, and failure rates) but also safety (such as the risk of infections, administration‐related reactions, and potential for exacerbating comorbidities), convenience for patients (e.g. hospital visit intervals, self‐injection, maintenance therapy at clinics, feasibility of drug discontinuation/readministration), and payment (medical costs) borne by patients.^8^

GUS, a fully human IgG1λ monoclonal against the p19 subunit of IL‐23 (IL‐23p19), has been shown to be effective and have a favorable safety profile in the treatment of psoriasis in previous clinical trials.^9^, ^10^, ^11^, ^12^, ^13^ In 2018, GUS was approved in Japan for the treatment of patients with psoriasis vulgaris (PsV), psoriatic arthritis (PsA), generalized pustular psoriasis (GPP) and erythrodermic psoriasis (EP), who have not adequately responded to conventional therapies. The ongoing postmarketing surveillance (PMS) study, which was required by the Pharmaceuticals and Medical Devices Agency (PMDA) as part of the Risk Management Plan and Pharmacovigilance Plan for GUS approval, has been planned and is being conducted to assess the safety and effectiveness of GUS in Japanese patients with psoriasis in clinical practice over a period of 52 weeks. This report presents results from the 20‐week interim analysis of the PMS for GUS in real‐world patients with psoriasis.

Although randomized controlled trials are generally considered the gold standard for evaluating the safety and efficacy of new treatments, strict inclusion and exclusion criteria often result in trial populations that are not representative of real‐world patients who may have comorbidities and use other concomitant medications.^14^ Therefore, real‐world studies are becoming increasingly important to provide evidence supporting treatment safety and effectiveness in actual clinical settings.^14^, ^15^ The limited body of prospective real‐world data currently available for GUS in patients with psoriasis highlights the importance of this study.^15^, ^16^, ^17^, ^18^, ^19^, ^20^

This interim analysis provides valuable insights into the characteristics of Japanese patients with psoriasis treated with GUS in real‐world clinical practice and early safety and effectiveness outcomes with GUS treatment. The results of this analysis are expected to inform ongoing discussions on the appropriate use of biologics for psoriasis in Japan.

## 2 METHODS

### 2.1 Study design

**FIGURE 1:**
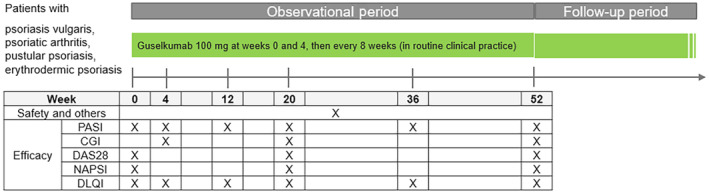
Study design. CGI, Clinical Global Impression; DAS28, Disease Activity Score in 28 Joints; DLQI, Dermatology Life Quality Index; NAPSI, Nail Psoriasis Severity Index; PASI, Psoriasis Area Severity Index.

This is a multicenter, single‐arm, prospective, observational longitudinal follow‐up cohort study in patients with PsV, PsA, GPP, and EP who initiated treatment with GUS in Japan. The observation period is 52 weeks after the initial dosing of GUS. GUS is administered by subcutaneous injection at a dose of 100 mg at weeks 0 and 4 followed by maintenance dosing with 100 mg every 8 weeks (Figure 1).

The protocol, including all ethical aspects, was submitted for internal review board approval and confirmed by PMDA. The study is conducted in accordance with the Japanese regulations for Good Post‐Marketing Study Practice, as specified by the Ministry of Health, Labour and Welfare Ministerial Ordinance no. 171. Initiation of the study occurred following establishment of a contract between the participating institutions and Janssen Pharmaceutical KK in Tokyo, Japan.

This is the interim report for a series of analyses of the ongoing study. This 20‐week interim analysis report presents results for patients who initiated treatment with GUS between May 2018 and October 2020.

### 2.2 Data collection and outcomes

Patient demographic and other baseline characteristics including medical history and comorbidities, as well as information pertaining to GUS treatment and concomitant drugs, were collected throughout the observation period. Investigators recorded adverse events (AEs), serious AEs, adverse drug reactions (ADRs; defined as AEs for which a causal relationship to drug treatment cannot be ruled out), and serious ADRs (SADRs) for safety assessments. AEs were coded by System Organ Class (SOC) and Preferred Term using Medical Dictionary for Regulatory Activities (MedDRA) version 25.0. Based on the Risk Management Plan for GUS, serious infections and serious hypersensitivity reactions were categorized as “important identified risks,” while malignancy, decreased neutrophil count, and major adverse cardiovascular events were classified as “important potential risks” for safety assessments.

The Psoriasis Area Severity Index (PASI), Clinical Global Impression (CGI) measure, which is evaluated by the treating clinician based on five potential responses (very much improved, much improved, minimally improved, no change, and worse),^21^ Dermatology Life Quality Index (DLQI),^22^ and Nail Psoriasis Severity Index (NAPSI)^23^ were used for assessment of effectiveness (Figure 1).

### 2.3 Statistical analysis

Safety evaluations were based on the safety analysis set, which included patients eligible for this study but excluded those with protocol violations, incomplete case report forms, and invalid case report forms. Effectiveness evaluations were based on the effectiveness analysis set, which consisted of patients in the safety set but excluded patients with missing or incomplete data on effectiveness evaluations.

Statistical analyses for the safety and effectiveness end points were performed on the safety analysis set and effectiveness analysis set, respectively. Change from baseline in PASI, DLQI, and NAPSI scores was assessed with the pairwise *t* test without multiplicity adjustment. Analyses were conducted to examine the influence of patient factors on PASI 90 response by estimating odds ratios and 95% confidence intervals using a logistic regression model, considering both crude (univariate) and adjusted (multivariate) values. In addition, the Wald test was used to calculate *P* values for odds ratios. Missing values were not imputed.

All statistical analyses were conducted using SAS version 9.4 (SAS Institute Inc.), in accordance with the user's manual.

## 3 RESULTS

### 3.1 Patient demographics and baseline characteristics

**FIGURE 2:**
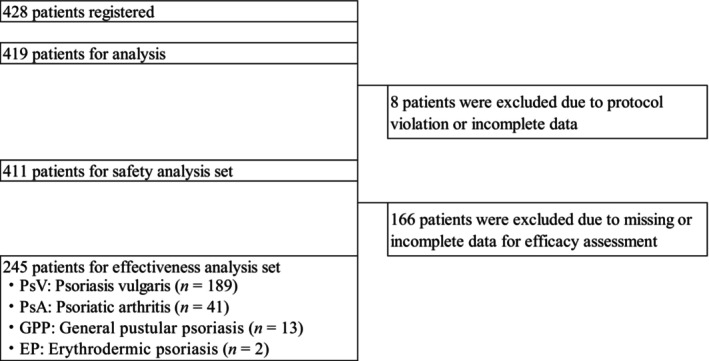
Disposition of study patients analyzed.

In total, 428 patients with psoriasis were registered during the enrollment period. Case report forms of 419 patients were available for the 20‐week interim analysis. Nine case report forms were uncollected. As eight patients with protocol violations and incomplete data were excluded, the safety analysis set consisted of 411 patients. The effectiveness analysis set comprised 245 patients, after excluding 166 patients from the safety analysis set due to incomplete data on effectiveness evaluations (Figure 2).

Table 1 summarizes the demographic and baseline characteristics of patients in the safety analysis set. There were more male patients (65.7%), and the mean age ± standard deviation was 57.8 ± 15.2 years. There were 152 (37.0%) elderly patients older than 65 years, with the oldest being 97 years. There were 307 (74.7%), 73 (17.8%), 26 (6.3%), and five (1.2%) patients who received GUS treatment for PsV, PsA, GPP, and EP, respectively. The mean duration of psoriasis was 13.9 ± 10.5 years. A total of 150 (36.5%) patients reported a disease duration of <10 years, and 84 patients had a disease duration of <5 years. The mean body surface area (BSA) was 22.7 ± 20.9% and 219 (53.3%) patients had a BSA > 10%. The mean PASI score was 11.7 ± 9.3 and 131 (31.9%) patients had a PASI score > 10. The majority of patients (85.4%) weighed less than 90 kg (mean body weight: 67.4 ± 14.7 kg). Most patients (83.7%) had previously received treatment with nonbiologic agents other than phototherapy; approximately half (46.7%) had received one or more prior biologic therapy for psoriasis. The most frequently used prior nonbiological oral treatment was apremilast (APR; 23.6%), while the most commonly used biologics were ustekinumab (UST; 22.1%), followed by secukinumab (SEC; 10.2%).

**TABLE 1:** Patient demographics and baseline characteristics

	*n* (%)
Number of safety analysis set	411
Sex	
Male	270 (65.7)
Female	141 (34.3)
Age (years)	
*n*	411
Mean ± SD	57.8 ± 15.2
<15	0 (0.0)
≥15 and <65	259 (63.0)
≥65	152 (37.0)
Body weight (kg)	
*n*	373
Mean ± SD	67.39 ± 14.65
Body mass index (kg/m^2^)	
*n*	372
Mean ± SD	24.90 ± 4.83
Primary disease	
Psoriasis vulgaris	307 (74.7)
Psoriatic arthritis	73 (17.8)
Pustular psoriasis	26 (6.3)
Psoriatic erythroderma	5 (1.2)
Disease duration (years)	
*n*	394
Mean ± SD	13.9 ± 10.5
<1	18 (4.4)
≥1 and <5	66 (16.1)
≥5 and <10	66 (16.1)
≥10 and <20	130 (31.6)
≥20 and <30	74 (18.0)
≥30	40 (9.7)
Missing	17 (4.1)
Body surface area at baseline (%)	
*n*	366
Mean ± SD	22.67 ± 20.92
<3	25 (6.1)
≥3 and ≤10	122 (29.7)
>10	219 (53.3)
Missing	45 (10.9)
PASI score at baseline	
*n*	270
Mean ± SD	11.65 ± 9.31
≤5	73 (17.8)
>5 and ≤10	66 (16.1)
>10 and ≤20	88 (21.4)
>20	43 (10.5)
Missing	141 (34.3)
Smoking history	
Current	71 (17.3)
Past	79 (19.2)
Never	126 (30.7)
Unknown	135 (32.8)
Medical history	
No	316 (76.9)
Yes	92 (22.4)
Missing	3 (0.7)
Comorbidity	
No	188 (45.7)
Yes	220 (53.5)
Missing	3 (0.7)
Prior biological treatment	
No	219 (53.3)
Yes	192 (46.7)
Prior biological treatment (agents)^a^	
Adalimumab	39 (9.5)
Infliximab	37 (9.0)
Ustekinumab	91 (22.1)
Secukinumab	42 (10.2)
Ixekizumab	26 (6.3)
Brodalumab	22 (5.4)
Other	4 (1.0)
Prior nonbiological treatment	
No	67 (16.3)
Yes	344 (83.7)
Prior nonbiological treatment (agents)^a^	
Cyclosporine	35 (8.5)
Retinoids	31 (7.5)
Steroids (oral)	15 (3.6)
Steroids (local)	258 (62.8)
Methotrexate	20 (4.9)
Apremilast	97 (23.6)
Other	49 (11.9)
Switched from prior biological treatment	
No	253 (61.6)
Yes	158 (38.4)
Switched from prior biological treatment (agents)^a^	
Adalimumab	12 (2.9)
Infliximab	15 (3.6)
Ustekinumab	73 (17.8)
Secukinumab	24 (5.8)
Ixekizumab	16 (3.9)
Brodalumab	14 (3.4)
Other	4 (1.0)
Swiched from prior nonbiological treatment	
No	314 (76.4)
Yes	97 (23.6)
Swiched from prior nonbiological treatment (agents)^a^	
Cyclosporine	11 (2.7)
Retinoids	5 (1.2)
Steroids (oral)	2 (0.5)
Steroids (local)	22 (5.4)
Methotrexate	5 (1.2)
Apremilast	56 (13.6)
Other	4 (1.0)

^a^ Duplicate case in a patient.

Abbreviations: PASI, Psoriasis Area Severity Index; SD, standard deviation.

Major comorbidities reported for study patients are presented in Table 2. Overall, 53.5% of patients had one or more comorbidity, including hypertension (20.9%), diabetes (10.9%), hyperuricemia (5.6%), dyslipidemia (4.9%), and hyperlipidemia (4.6%). Among six patients with hepatitis B virus (HBV), five had HBV‐DNA levels below the limit of quantification (one patient was untested) and one had received a DNA analog before starting GUS because of a positive hepatitis B antigen test. No patient had active tuberculosis (TB). Regarding latent TB, among 324 patients who underwent either a tuberculin reaction test or an interferon‐γ release test, eight patients were found to be positive for the tuberculin test, while six tested positive for the interferon‐γ release test. In addition, five patients in each group had received prophylactic anti‐TB drugs. Among the 239 patients for whom chest computed tomography scans were performed, no evidence of current or past TB infection was found.

**TABLE 2:** Common comorbidities (*N* = 411)

Comorbidities	*n* (%)
Hypertension	86 (20.9)
Diabetes	45 (10.9)
Hyperuricemia	23 (5.6)
Dyslipidemia	20 (4.9)
Hyperlipidemia	19 (4.6)
Chronic kidney disease	12 (2.9)
Hepatic steatosis	10 (2.4)
Interstitial lung disease	10 (2.4)
Osteoporosis	10 (2.4)
Gastrooesophageal reflux disease	8 (1.9)
Asthma	7 (1.7)
Depression	7 (1.7)
Angina pectoris	6 (1.5)
Gout	6 (1.5)
Gastric ulcer	6 (1.5)
Rheumatoid arthritis	6 (1.5)
Hepatitis B virus	6 (1.5)
Tinea pedis	6 (1.5)
Insomnia	5 (1.2)
Sleep apnea syndrome	5 (1.2)
Benign prostatic hyperplasia	5 (1.2)

### 3.2 Safety

The incidence of ADRs over the 20‐week analysis period is presented in Table 3. Overall, ADRs were reported for 27 (6.6%) patients, including nine (2.2%) patients with SADRs in the safety analysis set (*n* = 411). The most commonly reported ADRs by SOC was “Infection and infestations” (2.4%), with nasopharyngitis being the most frequently observed infection (0.7%). The next most frequent ADRs by SOC was “Skin and subcutaneous tissue disorders” (2.2%), with urticaria (0.7%) being the most common ADR. Other ADRs by SOC observed in ≥0.5% of patients included “Respiratory, thoracic and mediastinal disorders” (1.0%), “Hepatobiliary disorders” (0.5%), and “Investigations” (0.5%). Overall, 10 SADRs were reported, including four serious infections (aspergilloma, herpes zoster, pneumonia, enteritis infectious) and one malignancy (hepatocellular carcinoma); other reported SADRs included interstitial lung disease, hyponatremia, emphysema, hepatic function abnormal, and exacerbation of erythrodermic psoriasis. No cases of TB relapse or HBV reactivation were reported. Furthermore, no cases of serious hypersensitivity reactions, neutrophil count decrease, or major adverse cardiovascular events were identified in the 20‐week interim analysis.

**TABLE 3:** Incidence of ADRs during the initial 20 weeks of the observation period (*N* = 411)

ADRs by System Organ Class classification	Serious ADRs, *n* (%)	All ADRs, *n* (%)
Total number of events	10	35
Total number of patients	9 (2.2)	27 (6.6)
*Infections and infestations*	4 (1.0)	10 (2.4)
Nasopharyngitis	0 (0.0)	3 (0.7)
Herpes zoster	1 (0.2)	2 (0.5)
Aspergilloma	1 (0.2)	1 (0.2)
Pneumonia	1 (0.2)	1 (0.2)
Enteritis infectious	1 (0.2)	1 (0.2)
Furuncle	0 (0.0)	1 (0.2)
Oral candidiasis	0 (0.0)	1 (0.2)
Tonsillitis	0 (0.0)	1 (0.2)
Bacterial infection	0 (0.0)	1 (0.2)
*Neoplasms benign, malignant and unspecified* (*incl cysts and polyps*)	1 (0.2)	1 (0.2)
Hepatocellular carcinoma	1 (0.2)	1 (0.2)
*Metabolism and nutrition disorders*	1 (0.2)	1 (0.2)
Hyponatraemia	1 (0.2)	1 (0.2)
*Respiratory, thoracic and mediastinal disorders*	2 (0.5)	4 (1.0)
Emphysema	1 (0.2)	1 (0.2)
Interstitial lung disease	1 (0.2)	1 (0.2)
Cough	0 (0.0)	1 (0.2)
Cough variant asthma	0 (0.0)	1 (0.2)
*Hepatobiliary disorders*	1 (0.2)	2 (0.5)
Hepatic function abnormal	1 (0.2)	1 (0.2)
Hyperplastic cholecystopathy	0 (0.0)	1 (0.2)
*Skin and subcutaneous tissue disorders*	1 (0.2)	9 (2.2)
Urticaria	0 (0.0)	3 (0.7)
Pruritus	0 (0.0)	2 (0.5)
Erythrodermic psoriasis	1 (0.2)	1 (0.2)
Alopecia	0 (0.0)	1 (0.2)
Pyoderma gangrenosum	0 (0.0)	1 (0.2)
Mechanical urticaria	0 (0.0)	1 (0.2)
*Musculoskeletal and connective tissue disorders*	0 (0.0)	1 (0.2)
Arthralgia	0 (0.0)	1 (0.2)
*General disorders and administration site conditions*	0 (0.0)	1 (0.2)
Pyrexia	0 (0.0)	1 (0.2)
*Investigations*	0 (0.0)	2 (0.5)
Blood creatine phosphokinase increased	0 (0.0)	1 (0.2)
Blood beta‐D‐glucan increased	0 (0.0)	1 (0.2)
Hepatic enzyme increased	0 (0.0)	1 (0.2)
KL‐6 increased	0 (0.0)	1 (0.2)

*Note*: MedDRA version 25.0. Safety data were classified by System Organ Class and Preferred Term.

Abbreviation: ADR, adverse drug reactions.

AEs leading to treatment discontinuation were reported for 10 patients, with six of these assessed as treatment related. Among the treatment‐related cases, four were SADRs (erythrodermic psoriasis, interstitial lung disease, pneumonia, and aspergilloma), while the other two cases (alopecia and pruritus) were nonserious. No deaths were reported through week 20.

### 3.3 Effectiveness

**FIGURE 3:**
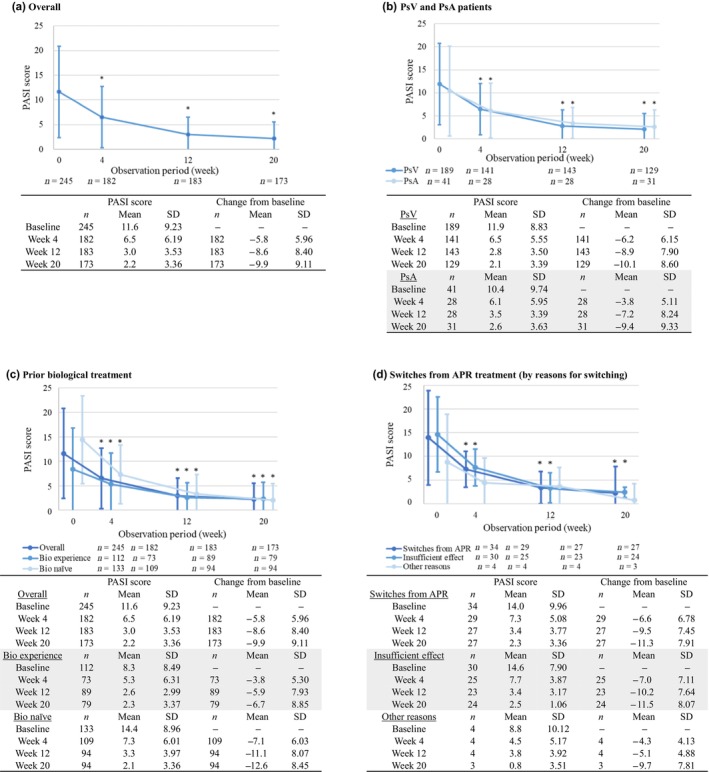
Psoriasis Area Severity Index (PASI) score (mean and standard deviation [SD]) at baseline and weeks 4, 12, and 20. (a) Overall. (b) Patients with psoriasis vulgaris (PsV) and psoriatic arthritis (PsA). (c) Patients with and without prior biological treatment. (d) Patients switched from apremilast (APR) treatment by reasons for switching. *p < 0.05 by pairwise *t*‐test for change from baseline PASI score by each time.

This analysis also aimed to evaluate the effectiveness of GUS in patients with psoriasis. For the effectiveness analysis population (*n* = 245), statistically significant decreases in mean PASI score from 11.6 at baseline to 6.5 at week 4 and to 2.2 at week 20 were observed (*p* < 0.001; Figure 3a). Significant improvements were observed for both PsV and PsA patient groups, with mean PASI scores of 11.9 and 10.4 at baseline, respectively, decreasing to 6.5 and 6.1 at week 4, and 2.1 and 2.6 at week 20 (Figure 3b). Moreover, although mean PASI scores at baseline were 8.3 and 14.4 for biologic‐experienced and biologic‐naive patients, respectively, both groups demonstrated significant improvements, with mean PASI scores of 5.3 and 7.3 at week 4, and 2.3 and 2.1 at week 20, respectively (Figure 3c).

A total of 181 patients switched from a nonbiologic treatment when initiating GUS, with a subset of 34 having switched APR. In addition, 89 patients switched from a prior biologic treatment. The primary reason for treatment switch was lack of efficacy among patients switching from either APR (88.2% [*n* = 30 of 34]) or a biologic (74.2% [*n* = 66 of 89]). Significant improvements in mean PASI score from baseline to each time point were observed for both patients switching from APR or a biologic, which is consistent with the trend observed in the overall patient population (*p* < 0.001; Figure 3d, Figure S1). A total of 15 patients who received cyclosporine (CyA) before starting GUS were evaluated separately; 10 patients discontinued CyA before starting GUS, whereas five patients continued CyA after initiating GUS. Comparable improvements in mean PASI scores were observed between the two groups (Figure S2).

**FIGURE 4:**
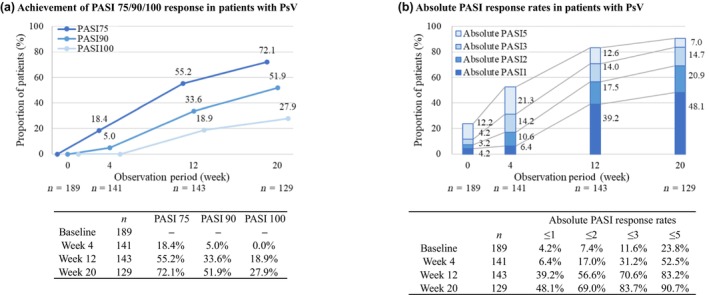
(a) Achievement of Psoriasis Area Severity Index (PASI) 75/90/100 response in patients with psoriasis vulgaris (PsV). (b) Absolute PASI response rates in patients with PsV.

Among patients with PsV (*n* = 189), the percentages of patients with PASI 75, 90, and 100 responses at week 20 were 72.1%, 51.9%, and 27.9%, respectively (Figure 4a). The response rates for achieving absolute PASI scores of ≤5, ≤3, ≤2, and ≤1 were 52.5%, 31.2%, 17.0%, and 6.4% at week 4 and 90.7%, 83.7%, 69.0%, and 48.1% at week 20, respectively (Figure 4b).

**FIGURE 5:**
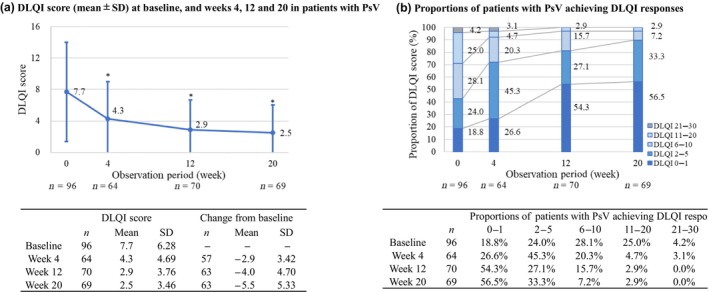
(a) Dermatology Life Quality Index (DLQI) score (mean and standard deviation [SD]) at baseline and weeks 4, 12, and 20 in patients with psoriasis vulgaris (PsV).*p < 0.05 by pairwise *t*‐test for change from baseline DLQI score by each time. (b) Proportions of patients with PsV achieving DLQI responses. A DLQI score level of 0 or 1 represents no effect of psoriasis on quality of life; 2 to 5, small effect; 6 to 10, moderate effect; 11 to 20, very large effect; and 21 to 30, extremely large effect.

The results depicted in Figure 5a demonstrate that mean DLQI score decreased significantly from 7.7 at baseline to 4.3 at week 4 and 2.5 at week 20. Moreover, by week 20, 56.5% of patients with PsV achieved a DLQI score ≤1, indicating no effect of psoriasis on quality of life. In addition, 89.9% of patients achieved a DLQI score ≤5, signifying either no effect or only a slight effect on their quality of life (Figure 5b).

**FIGURE 6:**
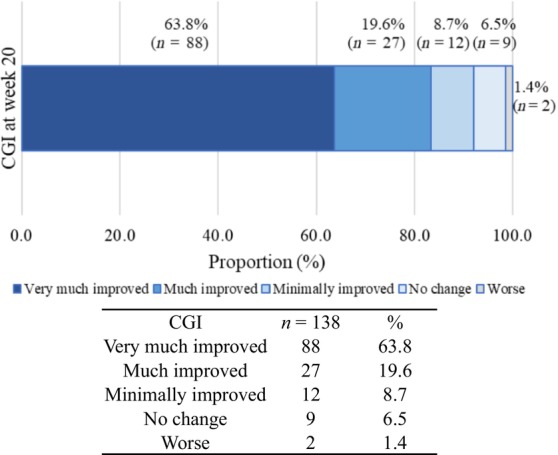
Clinical Global Impression (CGI) assessments at week 20.

Patients who were determined to be “very much improved,” “much improved,” or “minimally improved” based on their CGI score were considered treatment responders. Among all patients in the effectiveness analysis set, the CGI response rates were 83.0% at week 4 and 92.0% at week 20 (Figure 6).

**FIGURE 7:**
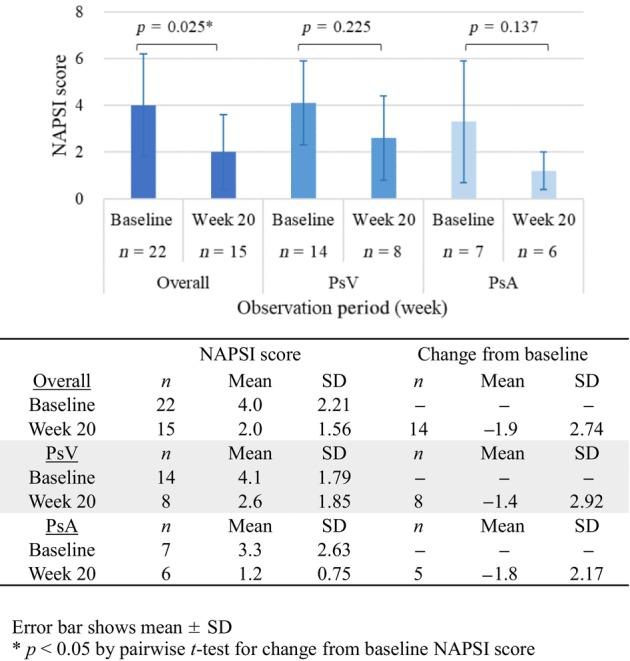
Nail Psoriasis Severity Index (NAPSI) score (mean ± SD) at baseline and week 20. PsA, psoriatic arthritis; PsV, psoriasis vulgaris.

Nail lesions were evaluated using the NAPSI. A clear decrease in mean NAPSI score from 4.0 at baseline to 2.0 at week 20 was observed for the overall study group. The severity of nail psoriasis was reduced for both the PsA and PsV patient subgroups, with notable improvements in mean NAPSI score from 3.3 and 4.1 at baseline to 1.2 and 2.6 at week 20, respectively (Figure 7).

Univariable and multivariable analyses (Figure S3) suggest that certain factors, such as a lower baseline PASI score and switching from a prior biologic treatment, were associated with a lower likelihood of achieving PASI 90.

## 4 DISCUSSION

This interim PMS analysis provides real‐world insights on the safety and effectiveness of GUS over the initial 20 weeks of treatment for patients with psoriasis in Japan. Real‐world studies, such as those for PMS purposes, are more likely to include elderly patients and those with prior treatment experience, who tend to be underrepresented in clinical trials.^18^, ^20^ Our study reflects this trend with a higher mean age of 57.8 years compared with 47.8 years in the Japanese Phase III PSO3004 trial for GUS in psoriasis.^12^ Moreover, our study found a higher percentage of patients (46.7%) with prior biologics experience compared with the PSO3004 trial, in which only 17.5% of patients reported biologics use. Differences in oral treatment history were also observed between these studies, with CyA having been previously used by 44.4% of PSO3004 trial patients, whereas our study reported much lower prior use of CyA among just 8.5% of patients. In addition, 23.6% of our study participants had ever previously used APR, which has become the most prescribed oral medication for psoriasis since 2017 based on an annual survey performed by the Japanese Society for Psoriasis Research.^24^ In the PSO3004 study, none of the patients in the GUS 100‐mg group had prior experience with APR because APR was approved after PSO3004 enrollment.

Variations in treatment trends and the transformative impact of biologics on psoriasis treatment over the past decade^24^ may account for disparities in prior treatment histories between the two studies. Furthermore, our study included a larger proportion of patients with previous treatment experience, including those who used more recently available biologics, potentially contributing to the lower baseline PASI score and BSA compared with the PSO3004 trial (mean PASI scores: 11.6 vs 26.7; mean BSA: 22.7% vs 37.9%). In addition, the inclusion of a pretreatment washout period in the PSO3004 trial may have led to the higher baseline PASI score and BSA compared with the real‐world PMS study. These findings underscore the value of real‐world data in gaining a comprehensive understanding of treatment outcomes in diverse patient populations.

In our study, we found that 53.5% of the enrolled patients had at least one comorbidity. The high prevalence of cardiovascular, metabolic, gastrointestinal, and chronic kidney disease comorbidities is aligned with findings reported in the existing literature.^24^, ^25^, ^26^ It is well‐known that these comorbidities may be associated with psoriasis severity; in particular, moderate to severe psoriasis may increase the risk of cardiovascular disease and mortality.^3^, ^27^, ^28^, ^29^, ^30^

PsA represents a major comorbidity associated with psoriasis typically preceding onset of joint symptom by several years in patients with PsA. In our PMS study 17.8% of patients received GUS for treating PsA. Recently, the concept of psoriatic disease (PsD) has been proposed, a systemic condition encompassing PsO and PsA, but also extends to include other key comorbidities such as metabolic syndrome, type 2 diabetes, and hypertension.^31^ A comprehensive approach is recommended to effectively address the multifaceted nature of PsD, which involves not only the treatment of cutaneous manifestations but also the holistic management of comorbidities and lifestyle modifications.^32^, ^33^, ^34^ Considering the prevalence of comorbidities in our study population and the concept of PsD management, we believe that close monitoring and early intervention using targeted therapeutics such as biologics may potentially have a positive impact on prognosis and prevention of new comorbidities,^35^, ^36^, ^37^, ^38^, ^39^ particularly in patients with high disease activity.^4^, ^40^ Adopting holistic and long‐term–oriented treatment strategies could further optimize the benefits for patients.

Our findings indicate that GUS was well‐tolerated and demonstrated favorable effectiveness outcomes comparable to those observed in other real‐world studies of GUS in psoriasis.^15^, ^16^, ^17^, ^18^, ^19^ No new safety signals were identified, and the incidence of ADRs was either comparable to or lower than previously reported rates.^9^, ^10^, ^11^, ^12^, ^13^, ^16^, ^18^ These findings further support the overall safety profile of GUS in real‐world settings.

In terms of effectiveness, our study demonstrated significant reductions in PASI score as early as 4 weeks and at 12 and 20 weeks after initiating GUS treatment. Significant improvements in PASI score were observed not only in the overall patient population but also in various subgroups, including those comprising patients with PsV, with PsA, with or without prior exposure to biologic therapies, as well as subgroups of patients who switched from APR or a biologic to GUS.

After 20 weeks of GUS treatment, mean PASI scores ranged from 2.1 to 2.6 across the above‐mentioned subgroups. These outcomes underscore the consistent effectiveness of GUS in treating psoriasis, regardless of variations in patient profiles. The efficacy of GUS across diverse patient subgroups has also been demonstrated in phase 3 trials of VOYAGE 1 (A Study of Guselkumab in the Treatment of Participants With Moderate to Severe Plaque‐Type Psoriasis) and VOYAGE 2 (A Study of Guselkumab in the Treatment of Participants With Moderate to Severe Plaque‐Type Psoriasis With Randomized Withdrawal and Retreatment).^41^ Moreover, in recent years, consideration of the absolute PASI scores has been increasingly recognized as an important measure for assessing treatment effectiveness in clinical practice. Achieving an absolute PASI score below the threshold of 2 or 3 holds considerable clinical importance, representing a concrete and clinically meaningful reduction in symptoms.^42^ Noticeably, our findings highlight how GUS treatment effectively enables the achievement of this high level of response, which is well‐aligned with established treatment goals.

In addition, the subgroup analysis provides new insights into the effectiveness of GUS among patients switching from APR. Previous reports^43^, ^44^ have indicated varying persistence rates for APR in real‐world use, with patients discontinuing due to perceived challenges in achieving desired effectiveness. Our findings support that GUS not only improves PASI scores among patients switching from APR but also provides a therapeutic advantage for patients exhibiting an inadequate response to APR.

The use of biologics in combination with other systemic therapies is controversial in clinical practice. JDA guidance advises against combining biologics with CyA.^8^ However, in some situations, CyA may be used in combination with a biologic, primarily due to concerns about temporary worsening of psoriasis. Our exploratory analysis investigated two CyA patient subgroups: one with patients maintaining CyA at the start of GUS treatment (five patients), and the other with patients who discontinued CyA before starting GUS (10 patients). Patients in both subgroups experienced notable decreases in PASI scores within 4 weeks, without any observed exacerbations. These findings suggest that, depending on the level of disease control, discontinuing CyA before initiating GUS might not have a substantial impact on maintaining efficacy.

In patients with PsV, we also evaluated PASI 90, absolute PASI, and DLQI responses. The PASI 90 response at 20 weeks in patients receiving GUS was 51.9% in our study, whereas a PASI 90 response rate of 77.8% was observed at 16 weeks in the PSO3004 trial. This observation aligns with findings from a previous real‐world psoriasis study.^45^ Lower PASI 90 response rates in real‐world clinical practice compared with clinical trials can be attributed to several factors. Our analysis of predictive factors for effectiveness showed that lower PASI 90 response rates were associated lower baseline PASI scores and switching from a prior biologic treatment. Of note, our study includes higher proportions of patients with a low baseline PASI score and/or prior biologic experience compared with the PSO3004 trial, which likely contributes to the observed difference in PASI 90 response rates between studies.

Rates for achieving absolute PASI scores of ≤2 or 3, which represent clinically meaningful improvements, at 4, 12, and 20 weeks were 17.0% or 31.2%, 56.6% or 70.6%, and 69.0% or 83.7%, respectively. Improvements in DLQI score were observed throughout the study, with around 90% of patients achieving a DLQI score of ≤5 at 20 weeks. These findings align with other real‐world studies demonstrating the overall positive impact of GUS treatment on the health‐related quality of life of patients with PsV.

In consideration of the findings reported here, certain limitations to the study must be acknowledged. In this observational study, there was no control group and it included patients with different types of psoriasis resulting in a heterogeneous study population. Further, the small proportion of patients with PsA, GPP, and EP limited analysis of each specific disease group in this interim analysis. In addition, this analysis was limited to a 20‐week evaluation period, and further long‐term analyses are necessary. Despite these limitations, the strengths of this study are its prospective design, multicenter approach, and large cohort of Japanese patients with psoriasis. This study benefited from the collaboration of more than 80 sites across Japan, and the demographic characteristics of the study population did not differ notably from those reported in a prior epidemiological study of Japanese patients with psoriasis.^24^ Therefore, the safety and effectiveness findings for GUS in this study could be considered representative for the broader psoriasis patient population in real‐world clinical practice in Japan.

In conclusion, this 20‐week interim analysis of the GUS PMS study in Japan found no new safety concerns. In addition, the favorable effectiveness profile of GUS in patients with psoriasis, including those who switched to GUS from APR and a biologic, was reaffirmed. However, longer‐term data from this ongoing study and other sources are needed to better understand the safety and effectiveness profile of GUS in Japanese patients with psoriasis.

## CONFLICT OF INTEREST STATEMENT

Y. Tada has received research grants, collaborative research funding, and speaker fees from Maruho Co., Ltd., Eisai Co., Ltd., Nippon Boehringer Ingelheim Co., Ltd., Janssen Pharma K.K., UCB Japan Co., Ltd., Mitsubishi Tanabe Pharma Corporation, Torii Pharmaceutical Co., Ltd., AbbVie GK, Kyowa Kirin Co., Ltd., Sun Pharma Japan Limited, Amgen K.K., Eli Lilly Japan K.K., Taiho Pharmaceutical Co., Ltd. And Bristol‐Myers Squibb K.K.; and has served as a consultant for Janssen Pharmaceutical K.K. K. Yamanaka has received speaker fees from Eli Lilly Japan K.K., Janssen Pharmaceutical K.K., AbbVie GK, Sanofi K.K., Eisai Co., Ltd., Nippon Boehringer Ingelheim Co., Ltd., Bristol‐Myers Squibb K.K., Teikoku Seiyaku Co., Ltd., Mitsubishi Tanabe Pharma Corporation, Maruho Co., Ltd., Novartis Pharma K.K., Torii Pharmaceutical Co., Ltd., Pfizer Japan Inc., Leo Pharma, Taiho Pharmaceutical Co., Ltd., Nippon Kayaku Co., Ltd., Sun Pharma Japan Limited, UCB Japan Co., Ltd., Daiichi Sankyo Company, Limited, Celgene Corporation, Kaken Pharmaceutical Co., Ltd., Nobelpharma Co., Ltd. And Kyowa Kirin Co., Ltd.; has received grants‐in‐aid for scientific research, scholarship grants from Japanese Society of Investigative Dermatology, Maruho Co., Ltd., Torii Pharmaceutical Co., Ltd., Taiho Pharmaceutical Co., Ltd., Sasaki Chemical Co., Ltd., Kaken Pharmaceutical Co., Ltd., Nippon Kayaku Co., Ltd., Nihon Pharmaceutical Co., Ltd., Sun Pharma Japan Limited and Nippon Boehringer Ingelheim Co., Ltd.; and has served as a consultant for Janssen Pharmaceutical K.K. The other authors are full‐time employees of Janssen Pharmaceutical K.K., Tokyo, Japan.

## Supporting information


Figures S1‐S3.
