# Supplementary material for: Safety and effectiveness of guselkumab in Japanese patients with psoriasis: 20‐week interim analysis of a postmarketing surveillance study
Source: J Dermatol. 2024 May 15;51(6):779–90. doi: 10.1111/1346-8138.17255 (PMC11484128; doi:10.1111/1346-8138.17255)
Supplement: Supplementary file 1 — Figures S1‐S3. [file JDE-51--s001.pdf]

Supplementary Figure 1. PASI score (mean ± SD) at baseline, and weeks 4, 12 and 20 for switches from biological treatment by reason for switching

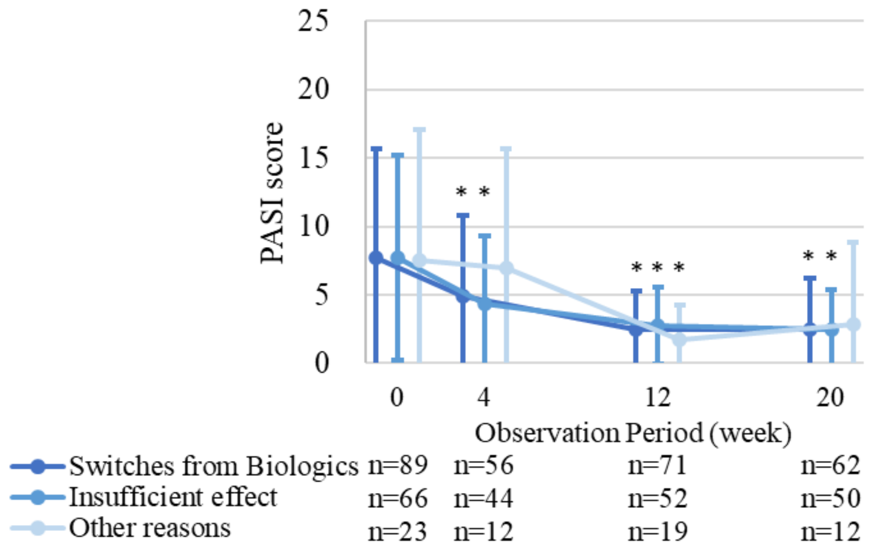

	PASI score			Change from baseline		
**Switches from biologics**	n	Mean	SD	n	Mean	SD
Baseline	89	7.7	8.00	-	-	-
Week 4	56	4.9	5.93	56	-3.6	5.58
Week 12	71	2.5	2.76	71	-5.3	7.66
Week 20	62	2.5	3.68	62	-5.7	8.19
**Insufficient effect**	n	Mean	SD	n	Mean	SD
Baseline	66	7.7	7.46	-	-	-
Week 4	44	4.4	4.92	44	-3.6	4.72
Week 12	52	2.7	2.80	52	-5.3	7.33
Week 20	50	2.4	2.97	50	-6.1	7.70
**Other reasons**	n	Mean	SD	n	Mean	SD
Baseline	23	7.5	9.55	-	-	-
Week 4	12	6.9	8.69	12	-3.5	8.29
Week 12	19	1.7	2.55	19	-5.2	8.69
Week 20	12	2.9	5.98	12	-3.8	10.14

Error var shows mean ± SD
* $p < 0.05$ by pairwise t-test for change from baseline PASI score by each time

Supplementary Figure 2. PASI score (mean ± SD) at baseline, and weeks 4, 12 and 20 by cyclosporine treatment

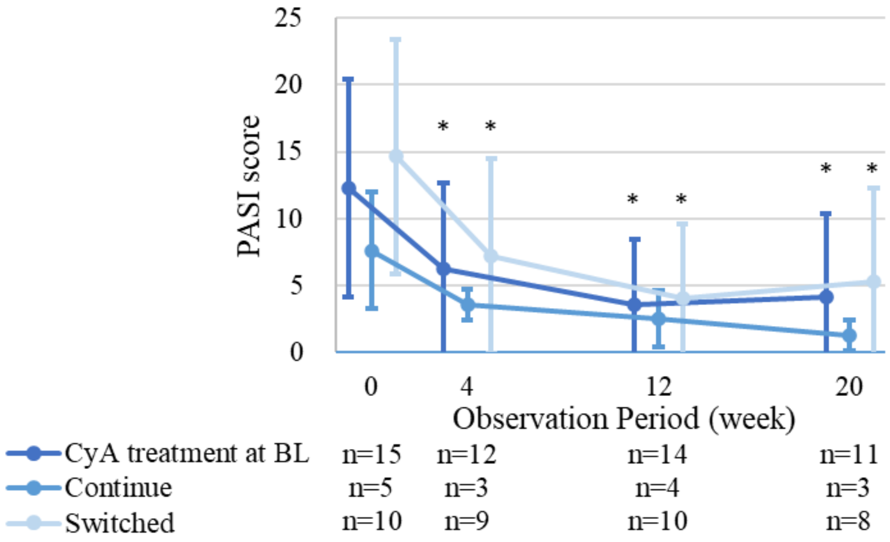

	PASI score			Change from baseline		
CyA treatment at BL	n	Mean	SD	n	Mean	SD
Baseline	15	12.3	8.14	-	-	-
Week 4	12	6.3	6.43	12	-5.7	4.93
Week 12	14	3.6	4.80	14	-9.3	6.93
Week 20	11	4.2	6.17	11	-8.9	6.25
Continue	n	Mean	SD	n	Mean	SD
Baseline	5	7.6	4.38	-	-	-
Week 4	3	3.5	1.16	3	-3.2	2.73
Week 12	4	2.5	2.13	4	-6.0	5.20
Week 20	3	1.3	1.14	3	-5.4	4.49
Switched	n	Mean	SD	n	Mean	SD
Baseline	10	14.6	8.74	-	-	-
Week 4	9	7.2	7.27	9	-6.6	5.32
Week 12	10	4.1	5.57	10	-10.6	7.31
Week 20	8	5.3	7.00	8	-10.2	6.54

Error var shows mean ± SD

* $p < 0.05$ by pairwise t-test for change from baseline PASI score

Supplementary Figure 3. Predictive factors associated with achieving PASI90 response

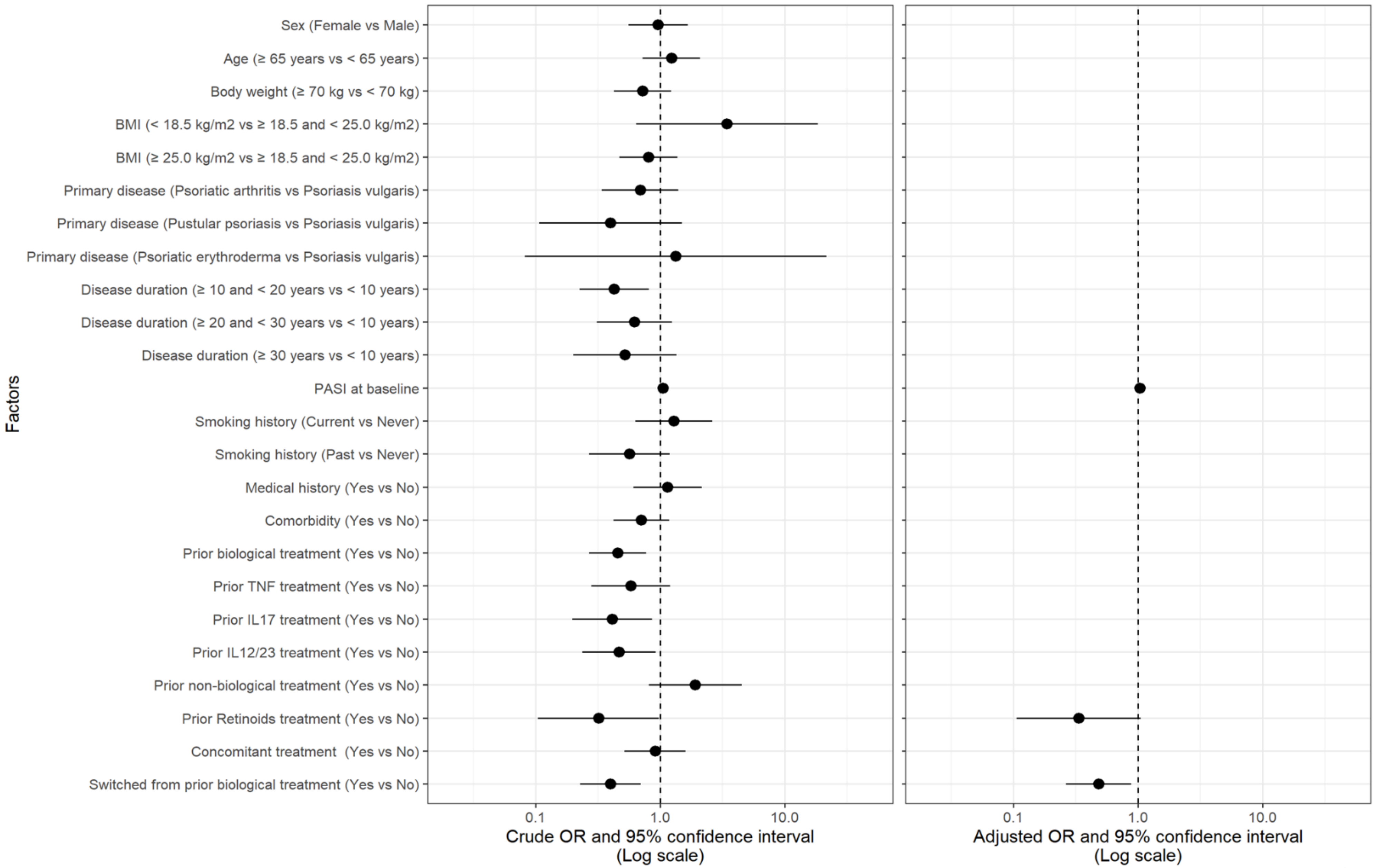

Factors	Crude odds ratio (95% CI)	Adjusted odds ratio (95% CI)
Sex (Female vs Male)	0.96 (0.56, 1.66)	
Age (≥ 65 years vs < 65 years)	1.23 (0.73, 2.10)	
Body weight (≥ 70 kg vs < 70 kg)	0.72 (0.43, 1.23)	
BMI (< 18.5 kg/m2 vs ≥ 18.5 and < 25.0 kg/m2)	3.44 (0.64, 18.43)	
BMI (≥ 25.0 kg/m2 vs ≥ 18.5 and < 25.0 kg/m2)	0.81 (0.47, 1.37)	
Primary disease (Psoriatic arthritis vs Psoriasis vulgaris)	0.69 (0.34, 1.40)	
Primary disease (Pustular psoriasis vs Psoriasis vulgaris)	0.40 (0.11, 1.50)	
Primary disease (Psoriatic erythroderma vs Psoriasis vulgaris)	1.33 (0.08, 21.64)	
Disease duration (≥ 10 and < 20 years vs < 10 years)	0.43 (0.23, 0.81)*	
Disease duration (≥ 20 and < 30 years vs < 10 years)	0.62 (0.31, 1.24)	
Disease duration (≥ 30 years vs < 10 years)	0.52 (0.20, 1.36)	
PASI at baseline	1.06 (1.03, 1.09)*	1.04 (1.01, 1.07)*
Smoking history (Current vs Never)	1.29 (0.63, 2.62)	
Smoking history (Past vs Never)	0.57 (0.27, 1.19)	
Medical history (Yes vs No)	1.15 (0.61, 2.17)	
Comorbidity (Yes vs No)	0.71 (0.42, 1.18)	
Prior biological treatment (Yes vs No)	0.46 (0.27, 0.77)*	
Prior TNF treatment (Yes vs No)	0.58 (0.28, 1.21)	
Prior IL17 treatment (Yes vs No)	0.41 (0.20, 0.86)*	
Prior IL12/23 treatment (Yes vs No)	0.47 (0.24, 0.92)*	
Prior non-biological treatment (Yes vs No)	1.91 (0.81, 4.50)	
Prior Retinoids treatment (Yes vs No)	0.32 (0.10, 0.98)*	0.33 (0.11, 1.05)
Concomitant treatment (Yes vs No)	0.91 (0.52, 1.60)	
Switched from prior biological treatment (Yes vs No)	0.40 (0.23, 0.70)*	0.48 (0.27, 0.88)*

* $p < 0.05$ by Wald test for logistic regression
